# Genetic Risk, Vascular Function, and Subjective Cognitive Complaints Predict Objective Cognitive Function in Healthy Older Adults: Results From the Brain in Motion Study

**DOI:** 10.3389/fnint.2020.571683

**Published:** 2020-11-03

**Authors:** Amanda V. Tyndall, R. Stewart Longman, Tolulope T. Sajobi, Jillian S. Parboosingh, Lauren L. Drogos, Margie H. Davenport, Gail A. Eskes, David B. Hogan, Michael D. Hill, Marc J. Poulin

**Affiliations:** ^1^Department of Physiology & Pharmacology, Cumming School of Medicine, University of Calgary, Calgary, AB, Canada; ^2^Hotchkiss Brain Institute, Cumming School of Medicine, University of Calgary, Calgary, AB, Canada; ^3^Department of Psychology, University of Calgary, Calgary, AB, Canada; ^4^Psychology Service, Foothills Medical Centre, Alberta Health Services, Calgary, AB, Canada; ^5^Department of Clinical Neurosciences, Cumming School of Medicine, University of Calgary, Calgary, AB, Canada; ^6^Department of Community Health Sciences, Cumming School of Medicine, University of Calgary, Calgary, AB, Canada; ^7^Department of Medical Genetics, Cumming School of Medicine, University of Calgary, Calgary, AB, Canada; ^8^Alberta Children’s Hospital Research Institute for Child and Maternal Health, University of Calgary, Calgary, AB, Canada; ^9^Departments of Psychiatry, Psychology and Neuroscience, Dalhousie University, Halifax, NS, Canada; ^10^Department of Medicine, Cumming School of Medicine, University of Calgary, Calgary, AB, Canada; ^11^Libin Cardiovascular Institute of Alberta, Cumming School of Medicine, University of Calgary, Calgary, AB, Canada; ^12^Faculty of Kinesiology, University of Calgary, Calgary, AB, Canada

**Keywords:** subjective memory complaints, cognitive aging, genetic risk, cerebrovascular circulation, objective cognitive function, exercise, Alzheiemer’s disease

## Abstract

Aging is associated with subjective memory complaints. Approximately half of those with subjective memory complaints have objective cognitive impairment. Previous studies have provided evidence of an association between genetic risk for Alzheimer’s disease (AD) and dementia progression. Also, aging is a significant risk factor for vascular pathology that may underlie at least some of the cognitive changes. This study investigates the relative contribution of subjective cognitive complaints (SCC), vascular function, and genetic risk for dementia in predicting objective cognitive performance. Multiple regression and relative importance analysis were used to investigate the relative contribution of vascular function, self-reported SCC, and dementia genetic risk, in predicting objective cognition in a sample of 238 healthy community-dwelling older adults. Age, sex, premorbid cognitive abilities, subjective verbal memory complaints, higher cerebrovascular blood flow during submaximal exercise, and certain dementia risk alleles were significant predictors of worse objective verbal memory performance (*p* < 0.001, *R*^2^ = 35.2–36.4%). Using relative importance analysis, subjective verbal memory complaints, and certain dementia risk alleles contributed more variance than cerebrovascular measures. These results suggest that age-related changes in memory in healthy older adults can be predicted by subjective memory complaints, genetic risk, and to a lesser extent, cerebrovascular function.

## Introduction

With typical cognitive aging, there is a decline in certain neurocognitive abilities and physiological functions (Blazer and Wallace, 2016). Although vocabulary and semantic knowledge are resistant to deterioration with aging, other neurocognitive skills such as memory, processing speed, and executive functions are more susceptible to decline with advancing age (Harada et al., 2013). There is substantial variability in the pace of cognitive aging, which appears to be influenced by genetic (McClearn et al., 1997), psychological (i.e., depression, anxiety, or personality; Slavin et al., 2010), vascular, and/or cerebrovascular factors (Brown et al., 2010; Ganguli et al., 2013), and medical comorbidities (Harada et al., 2013). With a projected increase in the number of older (65 years of age and older; 65+) adults to 1.5 billion worldwide by 2050 (Suzman and Beard, 2011), a better understanding of the cognitive changes associated with normal aging and those associated with pathological entities such as Alzheimer’s disease (AD) and related dementias, is of critical importance.

Non-demented individuals with subjective cognitive complaints (SCC) are made up of individuals with perceived cognitive decline but no objective evidence of cognitive impairment described as having subjective cognitive decline (SCD; Jessen et al., 2014) and persons with Mild Cognitive Impairment (MCI) where there are objective cognitive deficits on testing (Sanford, 2017). It is estimated that about half of non-demented, community-dwelling individuals with SCC have SCD while the other half have MCI (Gallassi et al., 2008). SCC without objective cognitive impairments may be explained by depressive symptoms and personality traits (e.g., neuroticism; Slavin et al., 2010), the inability of neuropsychological tests to detect subtle changes in objective cognitive function, especially in high functioning individuals (Jessen et al., 2014), and/or the use of differing criteria for detecting SCC (Reid and MacLullich, 2006). While these changes in cognitive function are annoying, it is important to note that most older adults will not develop cognitive impairment, let alone dementia (Harada et al., 2013).

Increasing age is associated with several physiological and anatomical brain changes that may be related to changes in cognitive functioning (Harada et al., 2013). Among these numerous changes, there is an estimated decline in cerebral blood flow (CBF) by approximately 5% per decade (Grolimund and Seiler, 1988). Cerebrovascular health may be of particular importance for cognitive function as CBF in older adults with SCC, MCI, and AD is lower than in healthy older adults (Binnewijzend et al., 2013). While CBF in healthy older adults is also lower than in young adults (Restom et al., 2007), the brain appears able to compensate for normal age-related decline in CBF. Restom et al. (2007) demonstrated this in healthy older adults who showed greater increases in functional CBF, as measured by quantitative arterial spin labeling, during memory encoding tasks than younger adults.

There is interest in identifying non-modifiable genetic risk factors associated with the development of Late-Onset Alzheimer’s Disease (LOAD; Lambert et al., 2009) and how these brain changes and genetic risk factors interact. Apolipoprotein (*APOE*) and its associated risk allele ε4, is the most studied gene related to LOAD. Carriers of one *APOE* ε4 allele have a 2.5–fold increased risk of LOAD, whereas carriers with two ε4 alleles have a 16-fold risk (Morgan, 2011). The most recent meta-analysis considered 11,632 single nucleotide polymorphisms and identified 11 loci with evidence of a significant association with the risk for LOAD (Lambert et al., 2013). Genetic variants with the greatest effects in the overall analysis were in *APOE*, bridging integrator 1 (*BIN1*), clusterin (*CLU*), complement receptor 1 (*CR1*), and phosphatidylinositol-binding clathrin assembly protein (*PICALM*, Lambert et al., 2013). Some of the proposed mechanisms by which genetic factors contribute to age-related cognitive decline involve cerebrovascular function (Tai et al., 2016). However, the precise mechanisms underlying the relationship between risk alleles and LOAD remain unclear with little known about the relative influences of LOAD genetic and non-genetic risk factors including SCC on objective cognitive performance.

Other researchers have found that SCC, increased age, sex (more complaints in females), lower premorbid IQ, and mood (i.e., more depressive symptoms) predict worse objective cognitive function (Jonker et al., 2000; Slavin et al., 2010). However, there is a gap in our knowledge of the contribution of cerebrovascular function and genetic risk for impaired cognition later in life on the relationship between objective cognitive function and SCC. The overarching aim of the manuscript was to determine how participant characteristics including cerebrovascular parameters, genetic factors, and SCCs are associated with objective cognitive outcomes. We had three *a priori* hypotheses: first, in a group of generally healthy middle-aged and older adults, there would be a relationship between SCC with objective cognitive performance; second, the greater LOAD genetic risk and the more impaired cerebrovascular function are, the worse will be objective cognitive performance; and, third, LOAD genetic risk and cerebrovascular function would explain more variance in the model predicting objective cognitive performance than the presence of SCCs.

## Materials and Methods

### Participants

This is a sub-study of the prospective cohort investigation called the *Brain in Motion* (BIM) study, which examined the effects of a 6-month aerobic exercise intervention on cerebrovascular and cognitive functions in sedentary, community-dwelling, and generally healthy older adults (aged 53–85 years; Tyndall et al., 2013). Participant recruitment and inclusion criteria are described in the Supplementary Material. For this sub-study, we used data from the pre-intervention baseline (Phase 1A) phase of BIM. All participants provided signed informed consent. The study was approved by the University’s Conjoint Health Research Ethics Board.

### Main Outcome Measure: Objective Cognitive Function

The neuropsychological assessment assessed seven composite cognitive domains: (1) processing speed; (2) concept formation; (3) verbal memory; (4) verbal fluency; (5) figural memory; (6) visual perceptual ability; and (7) complex attention (Supplementary Table 1). Composite domain scores were calculated by averaging Z-scores from each cognitive test. The composite cognitive domains were determined using a combination of exploratory and confirmatory factor analysis. All items in each factor had to meet the threshold of 0.4 for inclusion in the final structure. A detailed description of the neuropsychological tests used in the BIM study has been published (Tyndall et al., 2013).

### Predictors of Objective Cognitive Function

#### Subjective Cognitive Complaints

SCC was measured using the Multiple Abilities Self-Report Questionnaire (MASQ; Seidenberg et al., 1994). Complaints were tabulated across five domains within the MASQ: (1) language; (2) visual-perceptual ability; (3) verbal memory; (4) visual memory; and (5) attention/concentration. A sixth domain was created by adding the verbal memory and the visual memory domains, to create a total memory domain. Participants were dichotomized into two groups based on the total percentage with cognitive complaints. Previous studies have demonstrated that in community-dwelling healthy older adults, the prevalence of SCC is approximately 10–20% (Jungwirth et al., 2004). To allow for the comparison of our sample to other community-based samples of older adults with SCC, we sought to have approximately 10–20% of participants in the complaints group for each domain of SCC (Jungwirth et al., 2004). We, therefore, assessed 1 or more complaints vs. no complaints in the subjective domains of language, visual perceptual ability, attention, and visual memory; and two or more vs. no or one complaints in verbal memory and total memory subjective domains.

#### The Measure of Cerebrovascular Function

The cerebrovascular function was assessed using a euoxic hypercapnia test and submaximal exercise [at 40% maximal aerobic capacity ($\dot{V}{\text{O}}_{2}\max$) and 65 Watts (W)] as described previously (Brown et al., 2010; Tyndall et al., 2013) and in the Supplementary Material. The main outcomes of the cerebrovascular function assessment were: (1) Mean arterial pressure [MAP; calculated as 1/3 (systolic blood pressure − diastolic blood pressure) + diastolic blood pressure] as measured by beat-by-beat using finger pulse photoplethysmography (Finometer, Finapres Medical Systems, Amsterdam, Netherlands); (2) CBF measurement of the mean peak blood flow velocity through the middle cerebral artery (MCA; as denoted by $\bar{V}\text{P}$) using transcranial Doppler ultrasound (TCD); and (3) cerebrovascular conductance (CVC; calculated as $\bar{V}\text{P}$/MAP). Definitions of $\bar{V}\text{P}$ and CVC are described in the Supplementary Material.

#### Genotyping

The methodology for genotyping is described in the Supplementary Material. All allele frequencies of the single nucleotide polymorphisms did not significantly differ from Hardy-Weinberg equilibrium. Genetic risk was based on the presence of carrying a risk allele. Those who were homozygous and heterozygous for the risk allele were considered carriers of the risk allele (scored as 1), and homozygous wildtype was considered non-carriers with no risk (scored as 0; Table 1).

**Table 1:** The number of individuals carrying each genotype and each risk category at each of the loci analyzed.

Gene	dbSNP	Genotype	*n*	Risk allele	*n*
*APOE*	rs429358	ε2/ε2	1	ε4 non-carriers (no risk = 0)	169
	rs7412	ε2/ε3	27	ε4 carriers (risk = 1)	69
		ε2/ε4	6		
		ε3/ε3	141		
		ε3/ε4	55		
		ε4/ε4	8	
*BIN1*	rs744373	A/A	117	G non-carriers (no risk = 0)	117
		A/G	107	G carriers (risk = 1)	121
		G/G	14		
*CLU*	rs11136000	A/A	34	G non-carriers (no risk = 0)	34
		A/G	120	G carriers (risk = 1)	204
		G/G	84		
*CR1*	rs6656401	G/G	150	A non-carriers (no risk = 0)	150
		G/A	82	A carriers (risk = 1)	88
		A/A	6		
*PICALM*	rs3851179	A/A	23	G non-carriers (no risk = 0)	23
		A/G	119	G carriers (risk = 1)	215
		G/G	96		

**Note. APOE*, apolipoprotein E; *BIN1*, bridging integrator 1; *CLU*, clusterin; *CR1*, complement receptor 1; *PICALM*, phosphatidylinositol-binding clathrin assembly protein*.

#### Premorbid IQ and Depressive Symptoms

Co-variates for this study included age, sex, premorbid IQ [North American Adult Reading Test (NAART; Uttl, 2002; Pavlik et al., 2006)], and depression scores (Profile of Mood States POMS; McNair et al., 1981). The North American Adult Reading Test (NAART) was administered to evaluate participants’ past intellectual functioning (Uttl, 2002). The NAART is insensitive to MCI and has been demonstrated to be a useful assessment of premorbid cognitive abilities (Pavlik et al., 2006). The POMS (McNair et al., 1981) contains 65 adjectives that were rated by participants based on a five-point scale. Six factors were scored (tension, vigor, depression, fatigue, anger, and confusion) using a mean score from each factor.

### Statistical Analysis

Descriptive statistics were used to summarize participants’ demographic (age, sex, premorbid IQ), vascular function ($\bar{V}\text{P}$, CVC, MAP), and SCC with matching neuropsychological test characteristics (Supplementary Table 2). All analyses were two-tailed, with statistical significance determined at α ≤ 0.05. Analyses were performed using SPSS 24.0 software (SPSS Inc., Chicago, IL, USA). The statistical procedure for each hypothesis of the study is outlined below.

#### Hypothesis 1: Relationship Between SCC and Objective Cognitive Performance

Composite cognitive domains of objective cognitive performance were mapped to each subjective domain recommended by the creators of the MASQ (Seidenberg et al., 1994). *T*-tests or appropriate non-parametric tests (i.e., *χ*^2^) were used (Supplementary Table 2) to compare differences between those with complaints and those without complaint. Multiple comparisons were corrected for using the Benjamini–Hochberg procedure (Hochberg and Benjamini, 1990). Univariate associations between SCC and objective composite cognitive domains were assessed using correlation.

#### Hypothesis 2: Determinants of Cognitive Function

For each of the seven composite cognitive domain (as listed above), multiple linear regression was used to assess the effect of LOAD risk genes, cerebrovascular function (MAP, $\bar{V}\text{P}$, or CVC at +1, during CO_2_ reactivity, 40% $\dot{V}{\text{O}}_{2}\max$ workload, or 65W workload), and each of the six domains of SCC to predict objective cognitive performance while controlling for participants’ age, sex (female coded 0, male coded 1), depression scores, and premorbid IQ (NAART). We assessed the normality assumption of linear regression by visual examination of the distribution of regression residuals. Model fit was assessed using model *R*^2^, the proportion of total variable in each composite cognitive domain explained by the explanatory variables. Visual examination of the residuals for each hierarchical linear regression model was used to meet the assumption of normality. Variance inflation factor (VIF) and conditional variance were used to assess multicollinearity among the predictors of objective cognitive performance. Predictors with high VIF and conditional variance were excluded from the models.

#### Hypothesis 3: Contribution of Variables on Objective Cognitive Performance

The relative importance of each independent variable in the multiple linear regression was determined using the Pratt index, which quantifies each explanatory variable’s contribution, as their relative contribution to model *R*^2^ (Thomas et al., 1998). This index ranges in value between 0 and 100%, with higher values indicating greater importance.

## Results

### Descriptive Characteristics

A total of 238 men and post-menopausal women (*M* = 65.6, *SD* = 6.3 years, 53.4% women) completed cognitive and physiological testing and consented to genetic testing (Table 2). Descriptive values for age, sex, premorbid IQ, depression scores, objective measure of cognition, and vascular variables, are shown for participants with and those without SCC (Supplementary Table 2). *T*-tests showed that those with verbal memory complaints had lower objective verbal memory scores [mean (SD) = −0.459 (1.156)] than those without these complaints [mean (SD) = 0.110 (0.856), *t*_(233)_ = 2.97, *p* < 0.05; Supplementary Table 2]. No other statistically significant differences were observed between SCC groups and objective cognitive function domains (*p* > 0.05).

**Table 2:** Participant characteristics, values represent means (*SD*).

Characteristic	*n*	*M (SD)*
Demographics		
Age (years)	238	65.6 (6.3)
Females (%)	127 (53.4)	
NAART (Score)	238	110.2 (6.8)
Education (years)	238	15.8 (2.7)
MoCA (Score)	238	27.6 (1.4)
Health measures		
Systolic blood pressure (mmHg)	238	125.4 (15.9)
Diastolic blood pressure (mmHg)	238	72.1 (9.0)
Mean arterial pressure (mmHg)	238	89.7 (10.4)
Weight (kg)	235	77.9 (14.6)
BMI (km/m^2^)	235	27.2 (3.8)
Physical fitness		
$\dot{V}{\text{O}}_{2}\max$ (ml/kg/min)	234	26.1 (5.7)
40% $\dot{V}{\text{O}}_{2}\max$ work rate (W)	238	51.6 (16.6)

*Note. BMI, Body mass index; MoCA, Montreal Cognitive Assessment; NAART, North American Adult Reading Test; $\dot{V}{\text{O}}_{2}\max$, Maximal aerobic capacity*.

### Hypothesis 1: Relationship Between SCC and Objective Cognitive Performance

Weak correlations were observed between objective cognitive performance and verbal memory, total memory, language, and visual perceptual MASQ domains (Supplementary Table 3). Therefore, we restricted *Hypothesis 2* (determinants of the objective function) to the relationship between subjective verbal memory complaints and the objective composite cognitive domains of verbal memory, concept formation, and verbal fluency, as these were the only domains showing a link between subjective and objective cognitive function. In addition for *Hypothesis 2*, we also assessed the relationship between subjective total memory complaints and the objective verbal memory composite cognitive domain.

### Hypothesis 2: Determinants of Objective Cognitive Function

Consistent with previous work (Nguyen et al., 2016), age (*r* = −0.226, *p* < 0.001), sex (*r* = −0.393, *p* < 0.001), and premorbid IQ (*r* = 0.286, *p* < 0.001) were associated with verbal memory and were used as co-variates in the analysis for this study. While depression scores were not significantly associated with verbal memory performance (*r* = −0.093, *p* = 0.154), there was a weak correlation with verbal memory complaints (*r* = 0.139, *p* = 0.033) and thus, we retained depression as a co-variate.

In multiple linear regression analyses, vascular measures taken at the 65W workload best predicted objective cognitive performance, after controlling for age, sex, premorbid IQ, depression, SCC, and LOAD genetic risk (Supplementary Table 4). Specifically, when adjusting for age, sex, and NAART, multiple linear regression models demonstrated that $\bar{V}\text{P}$ (*R*^2^ = 0.352; *F*_(11,215)_ = 10.613; *p* < 0.001) and CVC (*R*^2^ = 0.359; *F*_(11,215)_ = 10.929; *p* < 0.001) were the best vascular predictors in the models showing a significant relationship between the subjective verbal memory domain and the objective verbal memory performance. Similarly, when adjusting for the same variables (age, sex, and NAART), multiple linear-regression models demonstrated that $\bar{V}\text{P}$ (*R*^2^ = 0.355; *F*_(11,214)_ = 10.702; *p* < 0.001) and CVC (*R*^2^ = 0.364; *F*_(11,214)_ = 11.120; *p* < 0.001) were the best vascular predictors for the relation between total memory complaints and objective total memory performance. Multiple linear regression models demonstrated that vascular variables (MAP, $\bar{V}\text{P}$, and CVC) measured at +1 mmHg CO_2_ or during the cerebrovascular reactivity test did not predict objective cognitive performance (i.e., *p* > 0.05). Similarly, multiple linear regression models demonstrated that vascular measures taken during the 40% $\dot{V}{\text{O}}_{2}\max$ workload had only a non-significant effect on objective cognitive performance (i.e., *p* > 0.05).

Verbal memory performance was the only objective composite cognitive domain predicted by all of our risk predictors including SCC (verbal memory and total memory subjective domains), vascular variables ($\bar{V}\text{P}$ and CVC) at 65W, and LOAD genetic risk (Tables s 3, 4). Both verbal and total memory complaints, older age, male sex, lower premorbid IQ, higher $\bar{V}\text{P}$ and CVC during submaximal exercise, *CLU* and *PICALM* risk, and *BIN1* no risk predicted lower verbal memory performance (Tables s 3, 4).

**Table 3:** Results from multiple regression analysis and relative contribution of predictors of verbal memory performance in BIM Study participants using verbal memory complaints.

	Verbal memory performance			
Predictor	Regression coefficients (SE)	*p*-value	Pratt index^a^ (%)	Model *R*^2^ (%)
Age	−0.040 (0.009)	<0.001	18.0	35.9^b^
Sex	−0.633 (0.101)	<0.001	39.5	
NAART	0.024 (0.008)	0.002	13.5	
CVC 65W	−0.973 (0.347)	0.006	2.4	
Verbal memory complaints	−0.428 (0.170)	0.013	7.9	
Depression	−0.002 (0.008)	0.782	0.4	
*APOE*	−0.041 (0.112)	0.713	0.6	
*BIN1*	0.311 (0.101)	0.002	7.3	
*CLU*	−0.298 (0.143)	0.038	5.1	
*CR1*	0.017 (0.105)	0.871	0	
*PICALM*	−0.485 (0.173)	0.006	5.3	
Age	−0.034 (0.008)	<0.001	14.8	35.2^c^
Sex	−0.713 (0.108)	<0.001	40.1	
NAART	0.024 (0.008)	0.002	13.2	
$\bar{V}\text{P}$ 65 W	−0.360 (0.153)	0.019	0	
Verbal memory complaints	−0.430 (0.171)	0.013	8.3	
Depression	−0.002 (0.008)	0.775	1.7	
*APOE*	−0.040 (0.112)	0.723	1.8	
*BIN1*	0.306 (0.101)	0.003	7.6	
*CLU*	−0.295 (0.143)	0.041	5.7	
*CR1*	0.032 (0.106)	0.761	1.3	
*PICALM*	−0.447 (0.174)	0.011	5.6	

*Note. APOE, apolipoprotein E; BIM, Brain in Motion; *BIN1*, bridging integrator 1; *CLU*, clusterin; *CR1*, complement receptor 1; CVC, cerebrovascular conductance (cm·s^−*1*^/mmHg); NAART, North American Adult Reading Test; *PICALM*, phosphatidylinositol-binding clathrin assembly protein; $\bar{V}\text{P}$, maximum peak systolic blood flow velocity (cm·s^−*1*^/mmHg). ^a^The Pratt index describes the contribution of each independent variable to the variance in the dependent variable rounded to the nearest tenth. ^b^Coefficient of multiple determination: *R*^2^ = 0.359; *F*_(11,215)_ = 10.929; *p* < 0.001. ^c^Coefficient of multiple determination: *R*^2^ = 0.352; *F*_(11, 215)_ = 10.613; *p* < 0.001*.

**Table 4:** Results from multiple regression analysis and relative contribution of predictors of verbal memory performance in BIM Study participants using total memory complaints.

	Verbal memory performance			
Predictor	Regression coefficients (SE)	*p*-value	Pratt index^a^ (%)	Model *R*^2^ (%)
Age	−0.041 (0.009)	<0.001	18.1	36.4^b^
Sex	−0.641 (0.101)	<0.001	40.3	
NAART	0.024 (0.007)	0.001	13.5	
CVC 65W	−0.971 (0.346)	0.005	2.6	
Total memory complaints	−0.293 (0.128)	0.023	5.4	
Depression	−0.003 (0.008)	0.723	0.5	
*APOE*	−0.082 (0.110)	0.454	1.2	
*BIN1*	0.329 (0.100)	0.001	8.0	
*CLU*	−0.308 (0.141)	0.030	5.2	
*CR1*	0.053 (0.105)	0.616	0	
*PICALM*	−0.470 (0.172)	0.007	5.1	
Age	−0.035 (0.008)	<0.001	15.1	35.5^c^
Sex	−0.715 (0.108)	<0.001	41.1	
NAART	0.025 (0.008)	0.001	13.4	
$\bar{V}\text{P}$ 65 W	−0.338 (0.153)	0.029	0	
Total memory complaints	−0.286 (0.129)	0.028	5.9	
Depression	−0.003 (0.008)	0.713	1.7	
*APOE*	−0.082 (0.110)	0.459	2.3	
*BIN1*	0.332 (0.101)	0.002	8.2	
*CLU*	−0.306 (0.142)	0.033	5.8	
*CR1*	0.068 (0.106)	0.518	1.2	
*PICALM*	−0.432 (0.173)	0.014	5.4	

*Note. APOE, apolipoprotein E; BIM, Brain in Motion; *BIN1*, bridging integrator 1; *CLU*, clusterin; *CR1*, complement receptor 1; CVC, cerebrovascular conductance (cm·s^−*1*^/mmHg); NAART, North American Adult Reading Test; *PICALM*, phosphatidylinositol-binding clathrin assembly protein; $\bar{V}\text{P}$, maximum peak systolic blood flow velocity (cm·s^−*1*^/mmHg). ^a^The Pratt index describes the contribution of each independent variable to the variance in the dependent variable rounded to the nearest tenth. ^b^Coefficient of multiple determination: *R*^2^ = 0.364; *F*_(11,214)_ = 11.120; *p* < 0.001. ^c^Coefficient of multiple determination: *R*^2^ = 0.355; *F*_(11,214)_ = 10.702; *p* < 0.001*.

### Hypothesis 3: Contribution of Variables on Objective Cognitive Performance

For each of the regression models, the Pratt index was used to rank the predictors according to their contributions to model *R*^2^ (Tables s 3, 4). These analyses revealed that sex was the most important predictor of objective cognitive function, explaining between 39.5% and 41.1% of the total variation explained by the regression model (i.e., total *R*^2^). Verbal memory complaints contributed to 7.9 and 8.3% of the *R*^2^ in the model predicting verbal memory performance with CVC and $\bar{V}\text{P}$ at 65W, respectively. In comparison, total memory complaints contributed to only 5.4 and 5.9% total prediction of verbal memory performance with CVC and $\bar{V}\text{P}$ at 65W, respectively. *CLU* and *PICALM* risk contributed approximately 5% to the models with CVC and $\bar{V}\text{P}$ at 65W. *BIN1* status contributed at least 7.3% and 8% of the *R*^2^ variance in the model predicting verbal memory performance with verbal memory complaints and total memory complaints, respectively. Vascular function variables, while significant within the models, contributed the least to the overall variance in the models predicting verbal memory performance.

## Discussion

The main finding of this study is that subjective verbal memory and total memory complaints predicted lower memory performance in participants, even after considering vascular measures, genetic risk, and demographics. As in previous studies, age, sex, and premorbid IQ were used as covariates in this study (Nguyen et al., 2016) and contributed the most to the total variation in objective verbal memory explained by the regression model (i.e., *R*^2^). In the overall models, age contributed to 15.8–18.2% of the variance in predicting verbal memory. Memory complaints have been observed to be one of the strongest predictors of reduced performance not only for memory but also for other aspects of cognition (Mol et al., 2006; Reid and MacLullich, 2006). Though the evidence is not entirely consistent, SCCs appear to predict a higher risk of future significant cognitive decline. In community-dwelling adults, the prevalence of memory complaints ranges from 22 to 52% among those 55+ (Mol et al., 2006) and increases to 88% among those 85+ (Reid and MacLullich, 2006). In the current study, subjective verbal memory complaints correlated with objective measures of concept formation, verbal memory, and verbal fluency (Supplementary Table 3). Exploratory analyses were conducted to assess the relationship between vascular measures, LOAD genetic risk, and verbal memory complaints on objective concept formation and verbal fluency. However, after controlling for these variables, verbal memory complaints did not predict objective concept formation or verbal fluency.

Previous studies have demonstrated that older adults who are more physically fit have higher cerebrovascular conductance (i.e., function) and better cognitive performance than their sedentary counterparts (Brown et al., 2010), suggesting a link between vascular function and cognitive health. Surprisingly, after adjusting for demographics, verbal memory complaints, and genetic risk, we observed that higher $\bar{V}\text{P}$ and CVC during the 65W workload predicted lower verbal memory. While this was an unexpected result, we did not see a relationship between CBF ($\bar{V}\text{P}$) for the cerebrovascular reactivity test or the 40% $\dot{V}{\text{O}}_{2}\max$ workload. These tests of cerebrovascular function may not have challenged the capacity of the vascular system enough to reveal any relationships with SCC and/or objective cognitive impairment. However, since the 65W workload was on average a harder work rate than a 40% $\dot{V}{\text{O}}_{2}\max$ workload, the relationship between higher blood flow and lower cognitive performance may have become evident for this condition because of the greater stress on cerebrovascular reserve capacity. While resting CBF decreases with advancing age (Grolimund and Seiler, 1988), an increase in CBF during a cognitive task may arise from compensatory adaptations to the lower resting CBF. We hypothesize that a decline in neural network structure or function may lead to changes in cognitive and physiological strategies that ultimately lead to altered function in specific brain regions (Cabeza et al., 2002). The brain may therefore increase activation of other areas (observed as an increase in CBF) to meet the demands of addressing a cognitive task (Cabeza et al., 2002; Park and Reuter-Lorenz, 2009). A study of resting CBF in presymptomatic carriers of the trinucleotide repeat expansion seen with Huntington’s disease showed a regional increase in resting CBF of the precuneus and the hippocampus regions (Wolf et al., 2011). However, a limitation of our study is that we could not look for regional differences in CBF with TCD. While uncertain that we were observing a compensatory response in cerebrovascular function specifically to submaximal exercise, it can be concluded (through the Pratt analysis) that the relative importance of vascular function was lowest, as it contributed the least in predicting verbal memory in this sample of healthy middle-aged and older adults.

While it was expected that age, sex, and premorbid IQ would contribute the most variance to the prediction of verbal memory performance (Jonker et al., 2000), the novel finding that *BIN1, CLU*, and *PICALM* contributed more relative importance to the models than vascular function during submaximal exercise is intriguing. After controlling for demographics, vascular function ($\bar{V}\text{P}$ and CVC), and verbal memory complaints, the risk alleles for *PICALM* and *CLU* predicted lower verbal memory performance. The proteins encoded by *CLU* and *PICALM* appear to play interactive roles as modifiers of Aβ toxicity in the brain (Zhang et al., 2015; Yang et al., 2016). With the greatest expression in neurons and endothelial cells of the blood-brain barrier (BBB), the translated PICALM protein regulates Aβ clearance through clathrin-mediated endocytosis and transcytosis across the BBB (Lambert et al., 2009; Zhao et al., 2015). The *CLU* gene encodes the protein clusterin, which is responsible for the conversion of Aβ into an insoluble molecule, thereby altering its toxicity (Lambert et al., 2009). *CLU* and *PICALM* risk variants affect memory specific brain regions (i.e., hippocampus) relatively early in life (Yang et al., 2016). Healthy young and older adults without MCI with the risk alleles for *PICALM* and *CLU* have smaller hippocampal volumes (Yang et al., 2016). It has been suggested that compensatory mechanisms such as increased CBF are needed to maintain normal brain function in carriers with these risk variants (Zhang et al., 2015). Functional compensation has been demonstrated in high-functioning older adults (Cabeza et al., 2002), but longitudinal studies of aging indicate that compensation may predict MCI or steeper trajectories of cognitive decline (Jessen et al., 2014). *CLU* and *PICALM* risk alleles appear to interact, at least in part, with cerebrovascular function, which may play a role in the different trajectories of age-related cognitive changes including changes in SCC.

In contrast to these genes, *BIN1* risk allele carriers had a better cognitive performance. The exact mechanisms by which tau pathology and B-amyloid accumulation are modified by the *BIN1* gene are not fully understood. While the *BIN1* risk allele has been identified as the second most prevalent susceptibility gene in GWAS studies, there are conflicting results presented in the literature on *BIN1* protein expression in Alzheimer’s disease and tau and B-amyloid pathology (Andrew et al., 2019). *BIN1* epigenetics is an important element in the pathogenesis of LOAD, and we propose that possibly the deleterious effects (i.e., tau pathology) may not have yet occurred in the healthy older adults of our study (Tan et al., 2013). In contrast, due to the small sample size for genetic analysis, we recognize that the *BIN1* risk group (the 121 G carriers) may have had by chance a worse profile for the other relevant characteristics measured in our study. Lastly, we did not see an effect of *APOE* or *CR1* on verbal memory (or any other composite cognitive domain) in our models. These results are similar to those of other studies that show no relationship between SCC and objective cognitive performance and *APOE* genotype in healthy older adults (Harwood et al., 2004).

A limitation of our study was that the cross-sectional design makes causal interpretation more difficult. An important follow-up component of the current study will assess the stability of cognitive complaints over time, and whether risk alleles continue to predict cognitive performance more strongly than vascular measures. This study was small for a study of genetic influences and should be repeated in a larger sample. While we were able to explain 35.2–36.4% of the variance in the analysis, other potentially important variables that were not examined in this study include sex steroid hormones, lipid profiles, and cognitive activities. Aside from the correlation between verbal memory complaints and objective verbal memory performance, we did not see a relationship between other domains of SCC and objective cognitive performance. This may be because the participants were high functioning and well-educated individuals. The cognitive performance of participants with SCC as indicated by mean z-scores was still within normal limits. Most differences were minor and of questionable clinical significance, as expected given the restricted sample. Previous research has identified subjective complaints as predicting a future decline, and this will be assessed on Follow-up. Future studies will evaluate the relationship between SSC, vascular function, and LOAD genetic risk to predict cognitive performance in larger samples of healthy older adults and those with MCI or dementia. Our conclusion that one predictor has more relative importance than another is limited by random variation in the Pratt index estimates. Consequently, these conclusions are only limited to the set of explanatory variables included in a regression model for this study and may not be generalizable to other populations. Future research will examine the validity of these findings in other external datasets.

In conclusion, this study investigated the contribution of vascular measures, genetic risk, and SCC in the prediction of objective cognitive performance. Overall, subjective memory complaints (both verbal and total), cerebrovascular function during submaximal exercise, and genetic risk (*PICALM, CLU*, and *BIN1*) significantly predict objective verbal memory. Further, in our sample, it appeared that genetic risk explains more of the variance in the model than vascular measures. We propose that the relationship between *CLU* and *PICALM* risk alleles and their effect on hippocampal morphology may influence cerebrovascular function through functional compensation mechanisms. Further, we suggest that the relationship between increased SCCs and worse objective cognitive functioning may be influenced by increased LOAD genetic risk and worse cerebrovascular function.

## Data Availability Statement

The original contributions presented in the study are publicly available. This data can be found here: https://prism.ucalgary.ca/handle/1880/112505.

## Ethics Statement

The studies involving human participants were reviewed and approved by University of Calgary Conjoint Health Research Ethics Board. The patients/participants provided their written informed consent to participate in this study.

## Author Contributions

The experimental design was conceived by GE, DH, MH, RL, and MP. MP takes full responsibility for the integrity of the data and the accuracy of the data analysis. MP led, guided, and oversaw data collection, validation, and final analyses. These data were collected by AT, LD, and MD. AT, RL, and TS contributed to the analysis and interpretation, and reviewed and edited the manuscript. JP provided the expertise, technical support, and supervision for the single nucleotide polymorphism determination, which was conducted by AT. Primary supervision for AT, LD, and MD was provided by MP. AT wrote the first draft of the manuscript and all co-authors contributed to the interpretation of the data and revision of the manuscript. All authors contributed to the article and approved the submitted version.

## Conflict of Interest

The authors declare that the research was conducted in the absence of any commercial or financial relationships that could be construed as a potential conflict of interest.
