# Supplementary material for: Genetic Risk, Vascular Function, and Subjective Cognitive Complaints Predict Objective Cognitive Function in Healthy Older Adults: Results From the Brain in Motion Study
Source: Front Integr Neurosci. 2020 Nov 3;14:571683. doi: 10.3389/fnint.2020.571683 (PMC7669615; doi:10.3389/fnint.2020.571683)
Supplement: Supplementary file 1 [file Table_1.docx]

Supplementary Material

# Supplementary Methods

## Participant recruitment and inclusion criteria

# Participants were recruited through media, poster, or newspaper advertisements. Inclusion criteria for potential study participants were: 1) absence of significant cognitive impairment (i.e., MCI or dementia) on a Montreal Cognitive Assessment score ≥ 24 (Nasreddine et al., 2005); 2) body mass index < 35kg/m^2^; 3) able to walk independently outside and up and down a flight of stairs: 4) no diagnosis of cardiovascular/cerebrovascular disease, neurological disorder, or obstructive sleep apnea; 5) non-smoker ≥12 months; 6) no major surgery in past six-months; and, 7) engagement in < 30 minutes moderate physical activity four days/week, or < 20 minutes vigorous activity two days/week (Tyndall et al., 2013).

## Genotyping

# Using standard protocols, genomic DNA was extracted from buffy coats of whole blood samples (Qiagen Gentra Puregene Blood Kit). The procedure for *APOE* (rs429358 and rs7412) genotyping has been previously described (Tyndall et al., 2016). In short, sequencing data were obtained by PCR-amplification followed by Sanger sequencing with specialized software (Mutation Surveyor DNA variant Analysis software, SoftGenetics, LLC, State Collage, PA) used to identify single nucleotide polymorphisms within the sequenced data. For *BIN1* (rs744373), *CLU* (rs11136000), *CR1* (rs6656401), and *PICALM* (rs3851179) TaqMan single nucleotide polymorphism assays (Applied Biosystems, Carlsbad, CA, USA) were used following standard protocols on StepOne Real Time PCR System and using Sequence Detection software for allelic discrimination.

## Measure of cerebrovascular function

# With their nose occluded by a nose clip, participants breathed room air through a mouthpiece attached to a fine capillary connected to a mass spectrometer (AMIS 2000, Innovision, Odense, Denmark) to collect end-tidal PCO_2_ and PO_2_ values. Cerebral blood flow was estimated using 2-MHz transcranial Doppler ultrasound (TCD) of the middle cerebral artery (MCA; Toc Neurovision^TM^, Multigon Industries, INC., Yonkers, NY). Resting end-tidal PCO_2_ and PO_2_ values were collected and averaged over a 10-minute air-breathing test (Chamber, University Laboratory of Physiology, Oxford, UK) to determine the end-tidal PCO_2_ and PO_2_ values for the euoxic hypercapnia test. The euoxic hypercapnia test consisted of one minute of room air breathing followed by stepped changes in end-tidal partial pressure of carbon dioxide (Petco_2_) to +1mmHg (5 minutes), +5mmHg (3 minutes), +8mmHg (3 minutes) and +1mmHg (5 minutes) above baseline values using the technique of dynamic end-tidal forcing and dedicated software (BreatheM v2.40, University Laboratory of Physiology, Oxford, UK) to accurately control desired Petco_2_ and Peto_2_ levels for each participant (Poulin et al., 1996).

# Following the euoxic hypercapnia test, participants were seated on a recumbent cycle ergometer breathing room air through a mouthpiece for a 30-minute protocol. The test consisted of six minutes of rest prior to five minutes of submaximal exercise at 50-60 revolutions per minute during which the cycle ergometer’s wattage (W) was set to 40% of each participant’s V̇O_2_max. (The assessment of V̇O_2_max was conducted on a separate day prior to the submaximal exercise test using techniques as previously described (Tyndall et al., 2013)). Following the 40% V̇O_2_max work rate was five minutes of rest, followed by five minutes of cycling exercise at an absolute work rate of 65W. The 65W work rate was chosen since it approximates an oxygen consumption of 1.0 L/min, and represents an oxygen demand for many activities of daily living (Passmore and Durnin, 1955). Data were collected every 10ms, averaged over each cardiac cycle (Poulin et al., 1998), and averaged again over the final 30 seconds of each stage.

# CVC is a measure of vascular tone, which is the change in after taking into consideration any change in MAP (Claassen et al., 2007). is a surrogate for cerebral blood flow which assumes that the axial flow velocity is proportional to laminar flow (for further discussion see (Tyndall et al., 2016)). As the cerebrovasculature is specifically sensitive to small changes in Petco_2_, was normalized to Petco_2_ ( /Petco_2_) (Spencer et al., 2015) and is referred to as throughout this paper. Cerebrovascular sensitivity to CO_2_, referred to as cerebrovascular reactivity, was calculated using a linear regression of the slope of the ∆ – ∆ Petco_2_ relationship to characterize the overall change across the stages of CO_2_ (i.e., +1mmHg, +5 mmHg, +8 mmHg) of the hypercapnia test.

**References**

Buschke, H., and Altman Fuld, P. (1974). Evaluating storage, retention and retrieval in disordered memory and learning. *Neurology* 24, 1019–1025. doi:​10.​1212/​WNL.​24.​11.​1019.

Claassen, J. A. H. R., Zhang, R., Fu, Q., Witkowski, S., and Levine, B. D. (2007). Transcranial Doppler estimation of cerebral blood flow and cerebrovascular conductance during modified rebreathing. *J. Appl. Physiol.* 102, 870–877. doi:10.1152/japplphysiol.00906.2006.

Delis, D. C., Kaplan, E., and Kramer, J. H. (2001). *Delis Kaplan Executive Functioning System (D-KEFS)*. San Antonio: NCS Pearson, Inc.

Hochberg, Y., and Benjamini, Y. (1990). More Powerful Procedures for Multiple Significance Testing. *Stat. Med.* 9, 811–818. doi:10.1002/sim.4780090710.

Lezak, M. (2004). *Neuropsychological Assessment*. New York: Oxford University Press.

Meador, K. J., Moore, E. E., Nichols, M. E., Abney, O. L., Taylor, H. S., Zamrini, E. Y., et al. (1993). The role of cholinergic systems in visuospatial processing and memory. *J. Clin. Exp. Neuropsychol.* 15, 832–842. doi:10.1080/01688639308402599.

Nasreddine, Z. S., Phillips, N. A., Bédirian, V., Charbonneau, S., Whitehead, V., Collin, I., et al. (2005). The Montreal Cognitive Assessment, MoCA: A brief screening tool for mild cognitive impairment. *J. Am. Geriatr. Soc.* 53, 695–699. doi:10.1111/j.1532-5415.2005.53221.x.

Passmore, R., and Durnin, J. (1955). Human energy expenditure. *Physiol. Rev.* 35, 801–840.

Poulin, M. J., Liang, P. J.-J., and Robbins, P. A. (1996). Dynamics of the cerebral blood flow response to step changes in end-tidal PCO2 and PO2 in humans. *J. Appl. Physiol.* 81, 1084–1095.

Poulin, M. J., Liang, P. J., and Robbins, P. A. (1998). Fast and slow components of cerebral blood flow response to step decreases in end-tidal PCO2 in humans. *J. Appl. Physiol.* 85, 388–397. doi:10.3109/11038129809035735.

Spencer, M. D., Tyndall, A. V, Davenport, M. H., Argourd, L., Anderson, T. J., Eskes, G. A., et al. (2015). Cerebrovascular Responsiveness to Hypercapnia Is Stable over Six Months in Older Adults. *PLoS One* 10, 1–17. doi:10.1371/journal.pone.0143059.

Stuss, D. T., Stethem, L. L., and Poirier, C. A. (1987). Comparison of three tests of attention and rapid information processing across six age groups. *Clin. Neuropsychol.* 1, 139–152. doi:10.1080/13854048708520046.

Tyndall, A. V., Argourd, L., Sajobi, T. T., Davenport, M. H., Forbes, S. C., Gill, S. J., et al. (2016). Cardiometabolic risk factors predict cerebrovascular health in older adults: Results from the Brain in Motion study. *Physiol. Rep.* 4. doi:10.14814/phy2.12733.

Tyndall, A. V., Davenport, M. H., Wilson, B. J., Burek, G. M., Arsenault-Lapierre, G., Haley, E., et al. (2013). The brain-in-motion study: Effect of a 6-month aerobic exercise intervention on cerebrovascular regulation and cognitive function in older adults. *BMC Geriatr.* 13, 1–10. doi:10.1186/1471-2318-13-21.

# Tables

**Supplementary Table 1:** Neuropsychological battery and specific tests used in each composite cognitive domain

| Composite Cognitive domain | Neuropsychological test | Measure |
| --- | --- | --- |
| Processing Speed | Symbol Digit Modalities (items matched) (Lezak, 2004) | Oral |
|  |  | Written |
|  | D-KEFS Color-Word Interference (s) (Delis et al., 2001) | Color naming |
|  |  | Word reading |
|  |  | Inhibition |
|  |  | Inhibition/switching |
| Concept Formation | D-KEFS Card Sorting (raw score) (Delis et al., 2001) | Number of sorts |
|  |  | Free sorting |
|  |  | Recognition |
| Verbal Memory | Buschke Selective Reminding Test (number of words recalled) (Buschke and Altman Fuld, 1974) | Summed learning |
|  |  | Delayed recall |
| Verbal Fluency | D-KEFS Verbal Fluency (number of words) (Delis et al., 2001) | Letter fluency |
|  |  | Category fluency |
|  |  | Category/switching |
| Figural Memory | MCG Figures Test (score) (Meador et al., 1993) | Immediate recall |
|  |  | Delayed recall |
| Visual Perceptual | MCG Figures Test (score) (Meador et al., 1993) | Copy trial |
| Complex Attention | Auditory Consonant Trigrams (summed score) (Stuss et al., 1987) | Total score |

*Note.* D-KEFS, Delis-Kaplin Executive Function System; MCG, Medical College of Georgia; s, seconds.

**Supplementary Table 2:** Participant characteristics, depression scores, matching objective cognitive performance to subjective cognitive complaint, and vascular outcomes for participants without complaints and those with cognitive complaints

|  | Language | | Visual perceptual | | Attention | | Visual memory | | Verbal memory | | Total memory | |
| --- | --- | --- | --- | --- | --- | --- | --- | --- | --- | --- | --- | --- |
|  | Without complaints | With complaints | Without complaints | With complaints | Without complaints | With complaints | Without complaints | With complaints | Without complaints | With complaints | Without complaints | With complaints |
| *n* | 194 | 44 | 189 | 49 | 185 | 52 | 181 | 55 | 211 | 25 | 186 | 49 |
| Age (years) | 65.6 (6.1) | 65.8 (7.1) | 65.6 (6.4) | 65.5 (5.9) | 65.9 (6.3) | 64.5 (6.1) | 65.4 (6.3) | 66.1 (6.4) | 65.4 (6.2) | 67.4 (6.5) | 65.3 (6.3) | 66.5 (6.2) |
| Sex (% female) ^a^ | 54.6 | 47.7 | 50.3 | 65.3 | 50.8 | 63.5 | 54.7 | 49.1 | 53.6 | 56.0 | 53.8 | 53.1 |
| NAART | 110.7 (6.2) | 107.6 (8.9) | 110.4 (6.9) | 109.5 (6.7) | 110.5 (6.6) | 109.1 (7.8) | 110.4 (6.4) | 109.4 (8.2) | 110.5 (6.3) | 107.0 (10.1) | 110.6 (6.2) | 108.3 (8.8) |
| Depression | 5.5 (6.5) | 8.0 (7.8) | 6.0 (7.0) | 5.9 (6.2) | 5.6 (5.9) | 7.4 (9.3) | 5.8 (7.2) | 6.6 (5.4) | 5.6 (6.7) | 8.7 (7.2) | 5.6 (6.9) | 7.1 (6.4) |
| Objective cognition  (z-scores) | 0.019 (0.804) | -0.048 (0.776) | 0.010 (1.040) | -0.113 (1.017) | -0.003 (0.244) | 0.008 (0.299) | 0.065 (0.956) | -0.172 (1.093) | 0.110 (0.856)* | -0.459 (1.156)* | 0.128 (0.854) | -0.213 (1.032) |
| MAP +1 (mmHg) | 89.9 (10.4) | 88.9 (10.2) | 90.1 (10.6) | 88.4 (9.6) | 89.6 (10.2) | 90.2 (11.1) | 90.3 (10.0) | 88.0 (11.7) | 90.0 (9.9) | 87.5 (13.9) | 90.3 (9.9) | 87.7 (12.1) |
| +1  (cm·s^-1^/mmHg) | 1.46 (0.31) | 1.51 (0.30) | 1.47 (0.31) | 1.46 (0.31) | 1.44 (0.29) | 1.58 (0.36) | 1.47 (0.29) | 1.49 (0.39) | 1.46 (0.31) | 1.52 (0.34) | 1.46 (0.29) | 1.52 (0.38) |
| CVC +1  (cm·s^-1^/mmHg) | 0.60 (0.16) | 0.64 (0.18) | 0.61 (0.17) | 0.60 (0.14) | 0.59 (0.16) | 0.66 (0.19) | 0.60 (0.16) | 0.62 (0.19) | 0.60 (0.16) | 0.66 (0.20) | 0.60 (0.16) | 0.64 (0.18) |
| MAP EX1 (mmHg) | 114.3 (16.4) | 111.3 (15.4) | 114.0 (17.1) | 113.1 (12.3) | 114.1 (15.6) | 112.0 (18.2) | 115.0 (15.8) | 109.0 (16.6) | 114.3 (15.7) | 110.0 (18.2) | 114.8 (15.5) | 109.8 (17.4) |
| EX1  (cm·s^-1^/mmHg) | 1.52 (0.33) | 1.59 (0.37) | 1.53 (0.34) | 1.54 (0.33) | 1.51 (0.33) | 1.63 (0.37) | 1.54 (0.34) | 1.54 (0.36) | 1.53 (0.33) | 1.61 (0.42) | 1.52 (0.33) | 1.61 (0.39) |
| CVC EX1  (cm·s^-1^/mmHg) | 0.51 (0.16) | 0.53 (0.16) | 0.51 (0.17) | 0.50 (0.13) | 0.50 (0.14) | 0.56 (0.21) | 0.50 (0.15) | 0.53 (0.19) | 0.51 (0.16) | 0.55 (0.16) | 0.50 (0.15) | 0.55 (0.19) |
| MAP EX2 (mmHg) | 119.9 (20.1) | 119.3 (16.9) | 119.1 (19.5) | 122.7 (19.7) | 120.3 (18.5) | 117.5 (23.2) | 121.3 (19.6) | 113.9 (18.3) | 120.0 (19.4) | 119.2 (19.2) | 120.4 (19.2) | 117.7 (20.0) |
| EX2  (cm·s^-1^/mmHg) | 1.54 (0.36) | 1.60 (0.35) | 1.54 (0.36) | 1.59 (0.36) | 1.52 (0.35) | 1.65 (0.37) | 1.54 (0.35) | 1.59 (0.41) | 1.54 (0.36) | 1.63 (0.41) | 1.52 (0.34) | 1.67 (0.43) |
| CVC EX2  (cm·s^-1^/mmHg) | 0.47 (0.16) | 0.48 (0.15) | 0.48 (0.17) | 0.46 (0.13) | 0.46 (0.13) | 0.53 (0.22) | 0.46 (0.15) | 0.51 (0.19) | 0.47 (0.16) | 0.49 (0.14) | 0.46 (0.15) | 0.50 (0.19) |

*Note.* Values represent mean (± *SD*). +1, values collected at +1mmHg Petco_2_; CVC, cerebrovascular conductance; EX1, values collected during 40% V̇O_2_max work rate; EX2, values collected during 65W work rate; MAP, Mean arterial pressure; NAART, North American Adult Reading Test; , maximum peak systolic blood flow velocity.

^a^ χ^2^ test used

**p* < .05 difference between with and without subjective cognitive complaints. T-tests corrected for multiple comparisons using Benjamini-Hochberg procedure (Hochberg and Benjamini, 1990).

**Supplementary Table 3:** Correlation of number of subjective cognitive complaints by MASQ domain and objective cognitive outcomes.

|  | | | Objective cognitive domain | | | | | |
| --- | --- | --- | --- | --- | --- | --- | --- | --- |
| MASQ Domain | Low complaints | High complaints | Processing Speed | Concept Formation | Verbal Memory | Verbal Fluency | Figural Memory | Complex Attention |
| Language | 0 (194) | ≥1 (44) | -.02 (234) | -.05 (238) | -.07 (237) | -.03 (238) | -.13* (238) | .06 (237) |
| Visual Perceptual | 0 (189) | ≥1 (49) | .10 (234) | -.07 (238) | .04 (237) | .10 (238) | -.16* (238) | .09 (237) |
| Attention | 0 (185) | ≥1 (52) | -.06 (233) | .09 (237) | .00 (236) | .01 (237) | -.10 (237) | .02 (236) |
| Verbal Memory | 0-1 (211) | ≥2 (25) | -.08 (232) | -.13* (236) | -.19** (235) | -.13* (236) | .09 (236) | -.10 (235) |
| Visual Memory | 0 (181) | ≥1 (55) | 0 (232) | .03 (236) | -.10 (235) | 0 (236) | -.10 (236) | -.03 (235) |
| Total Memory | 0-1 (186) | ≥2 (49) | -.01 (231) | -.05 (235) | -.15* (234) | -.09 (235) | .01 (235) | -.01 (234) |

*Note.* MASQ, Multiple Abilities Self-Report Questionnaire

Values represent Pearson correlation coefficient and sample size, *r* (*n*).

**p* < .05, ***p* < .01

**Supplementary Table 4:** Multiple linear-regression models for the relation between each domain of subjective cognitive complaint and accompanying objective cognitive outcome. Multivariable analysis adjusted for age, sex, NAART, vascular outcome (MAP, CVC, and ) at 65W workload, genetics (*APOE, BIN1, CLU, CR1, PICALM*).

| Predictor | | Outcome Variable | Regression Coefficients (SE) | *p*-value | Coefficient of multiple determination |
| --- | --- | --- | --- | --- | --- |
| Attention complaints | | Complex Attention |  |  |  |
|  | MAP (mmHg) |  | 0.011 (0.042) | 0.793 | *R*^2^ = .091; *F* _[11, 218]_ =1.973; *p* = .032 |
|  | (cm/s) |  | 0.015 (0.042) | 0.713 | *R*^2^ = .089; *F* _[11, 216]_ =1.923; *p =* .038 |
|  | CVC (cm·s^-1^/mmHg) |  | 0.014 (0.042) | 0.740 | *R*^2^ = .087; *F* _[11, 216]_ =1.862; *p* = .046 |
| Language complaints | | Verbal Fluency |  |  |  |
|  | MAP (mmHg) |  | 0.117 (0.126) | 0.354 | *R*^2^ = .242; *F* _[11, 220]_ =6.402; *p* < .001 |
|  | (cm/s) |  | 0.111 (0.127) | 0.384 | *R*^2^ = .241; *F* _[11, 218]_ =6.297; *p* < .001 |
|  | CVC (cm·s^-1^/mmHg) |  | 0.114 (0.126) | 0.366 | *R*^2^ = .242; *F* _[11, 218]_ =6.330; *p* < .001 |
| Visual perceptual complaints | | Visual perceptual |  |  |  |
|  | MAP (mmHg) |  | -0.077 (0.168) | 0.648 | *R*^2^ = .104; *F* _[11, 220]_ =2.331; *p* = .010 |
|  | (cm/s) |  | -0.081 (0.170) | 0.634 | *R*^2^ = .097; *F* _[11, 218]_ =2.133; *p* = .019 |
|  | CVC (cm·s^-1^/mmHg) |  | -0.064 (0.170) | 0.705 | *R*^2^ = .104; *F* _[11, 218]_ =2.312; *p* = .011 |
| Visual memory complaints | | Visual Memory |  |  |  |
|  | MAP (mmHg) |  | -0.281 (0.158) | 0.077 | *R*^2^ = .104; *F* _[11, 218]_ =2.295; *p* = .011 |
|  | (cm/s) |  | -0.262 (0.157) | 0.097 | *R*^2^ = .100; *F* _[11, 216]_ =2.192; *p* = .016 |
|  | CVC (cm·s^-1^/mmHg) |  | -0.255 (0.158) | 0.107 | *R*^2^ = .100; *F* _[11, 216]_ =2.193; *p* = .016 |
| Verbal memory complaints | | Verbal Memory |  |  |  |
|  | MAP (mmHg) |  | -0.440 (0.173) ^b^ | 0.012 | *R*^2^ = .333; *F* _[11, 217]_ =9.835; *p* < .001 |
|  | (cm/s) |  | -0.430 (0.171) ^ab^ | 0.013 | *R*^2^ = .352; *F* _[11, 215]_ =10.613; *p* < .001 |
|  | CVC (cm·s^-1^/mmHg) |  | -0.428 (0.170) ^ab^ | 0.013 | *R*^2^ = .359; *F* _[11, 215]_ =10.929; *p* < .001 |
| Total memory complaints | | Verbal Memory |  |  |  |
|  | MAP (mmHg) |  | -0.342 (0.128) ^b^ | 0.008 | *R* ^2^= .341; *F* _[11, 216]_ =10.183; *p* < .001 |
|  | (cm/s) |  | -0.286 (0.129) ^ab^ | 0.028 | *R*^2^ = .355; *F* _[11, 214]_ =10.702; *p* < .001 |
|  | CVC (cm·s^-1^/mmHg) |  | -0.293 (0.128) ^ab^ | 0.023 | *R*^2^ = .364; *F* _[11, 214]_ =11.120; *p* < .001 |

*Note.* CVC, cerebrovascular conductance; MAP, Mean arterial pressure; , maximum peak systolic blood flow velocity.

^a^ Cognitive complaint significant in predicting objective cognitive performance in model

^b^ One or more risk genes significant in predicting objective cognitive performance in model
